# Using deep-learning to forecast the magnitude and characteristics of urban heat island in Seoul Korea

**DOI:** 10.1038/s41598-020-60632-z

**Published:** 2020-02-26

**Authors:** Jin Woo Oh, Jack Ngarambe, Patrick Nzivugira Duhirwe, Geun Young Yun, Mattheos Santamouris

**Affiliations:** 1 Department of Architectural Engineering, Kyung Hee University, Yongin, 17104 Republic of Korea; 2 High Performance Architecture, School of Built Environment, University of New South Wales, Sydney, NSW 2052 Australia

**Keywords:** Environmental impact, Atmospheric science, Climate change

## Abstract

Urban heat island (UHI), a phenomenon involving increased air temperature of a city compared to the surrounding rural area, results in increased energy use and escalated health problems. To understand the magnitude and characteristics of UHI in Seoul and to accommodate for the high temporal variability and spatial heterogeneity of the UHI which make it inherently challenging to analyze using conventional statistical methods, we developed two deep learning models, a temporal UHI-model and a spatial UHI model, using a feed-forward deep neural network (DNN) architecture. Data related to meteorological elements (e.g. air temperature) and urban texture (e.g. surface albedo) were used to train and test the temporal UHI-model and the Spatial UHI-model respectively. Also, we develop and propose a new metric, UHI-hours, that quantifies the total number of hours that UHI exists in a given area. Our results show that UHI-hours is a better indicator of seasonal UHI than the commonly used index, UHI-intensity. Consequently, UHI-hours is likely to provide a better measure of the cumulative effects of UHI over time than UHI-intensity. UHI-hours will help us to better quantify the effect of UHI on, for example, the overall daily productivity of outdoor workers or heat-related mortality rates.

## Introduction

The world’s population is increasing dramatically; new cities are being built, while existing cities are getting overpopulated. Currently, more than 50% of the world population resides in cities^1^. This increased development in urban areas has resulted in changes in urban morphology and surfaces, as well as an increase in the amount of anthropogenic heat released to the atmosphere^2^. The combined effect of such changes leads to higher air temperature in urban areas than in the surrounding rural or suburban areas, hence the formation of heat islands^3^. These heat islands, especially in summers, considerably decrease the outdoor air quality and trigger heat-related diseases and deaths in urban areas^4,5^. Moreover, extreme temperatures have been reported to be the leading cause of weather-related deaths^6^. This is particularly prevalent among the elderly (people of 65 years of age and above) and those with cardiovascular and respiratory health issues. For example, Paravantis *et al*.^7^ analyzed the impact of temperature and heat waves on the number of deaths caused by cardiovascular and respiratory health issues in people above 65 years of age in Athens, Greece. They reported a U-shaped exposure-response curve, indicating reduced mortality rates at moderate temperatures and 20% and 35% increase in mortality rates at very low and very high temperatures, respectively. Furthermore, due to the increase in urban temperatures, there has been an increase in air-conditioning system usage, which in turn has led to high electricity demands and overall increased building energy consumption^8^. At the same time, passive cooling methods, such as natural and night ventilation, have become ineffective for the thermal comfort of building occupants^9^.

Due to the effects of urban heat island (UHI), the UHI issue has been receiving increased attention from many academic disciplines. A great part of this attention has been directed to the development of data-driven UHI-predictive models that can forecast UHI at a given time in the future. Such models are helpful in the development of UHI-mitigation strategies and in the drafting of relevant countermeasure policies. In addition, data-driven methods offer reduced computational load in the assessment of UHI than computer simulation programs such as UMEP-SOLWEIGH^10^, ENVI-MET^11^ etc., that are often used to model UHI behavior. Moreover, the process involved in conducting computer simulations is highly dependent on expert knowledge of the issue at hand. This makes the use of computer simulations in the analysis of UHI susceptible to errors in instances when the modeler lacks sufficient expert knowledge. Additionally, attaining an accurate model of complex urban geometries through analytical modeling requires a bulk of urban details that may not always be available to the casual user or difficult to obtain. In such cases, data-driven models provide a quick way to assess UHI behavior based on information that is commonly available and easily obtained (e.g. meteorological data). Moreover, data-driven models can be easily deployed in a system consisting of already programmed sensors (e.g. temperature sensors) to automatically and continuously display UHI levels at a given time based on prevailing environmental conditions and without human intervention.

Some of the common approaches used in developing UHI-predictive models include conventional regression methods. For instance, Chun and Guldmann^12^ explored urban UHI determinants using regression analysis and spatial data obtained from 2D- and 3D-aerial satellite images. Similarly, Su *et al*.^13^ demonstrated the relations among land-cover types (e.g., built-up, water, paddy field, and vegetation) using weighted-regression modeling. However, most regression methods are unable to capture complex nonlinear relations, which are also highly variable and spatially heterogeneous, between UHI determinants and UHI. Additionally, although there exist statistical methods that can be used to map non-linear data (e.g. non-linear regression methods), they often require iterative processes of fitting different types of mathematical functions to determine one that best fits a given dataset.

Consequently, advanced non-linear analysis techniques such as artificial neural networks (ANNs) have been extensively applied in studying the UHI phenomenon. With ANN algorithms, a model is automatically generated through the adjustment of weights and biases from one layer to another. For example, Mihalakakou *et al*.^14^ developed a backpropagation ANN model to estimate diurnal and nocturnal UHI intensity fluctuations in the Greater Athens area. This model is based on hourly temperature and solar radiation data collected from 23 automatic weather stations (AWSs) developed over a period of 2 years. The study identified urban air temperature as the predominant factor explaining nighttime and daytime UHI. In a different study, Mihalakakou *et al*.^15^ developed backpropagation ANN models based on synoptic-scale atmospheric circulation, ambient temperature, maximum daily solar radiation, and mean daily wind speed. Their conclusions emphasized the impact of synoptic-scale atmospheric circulation on UHI magnitude. Similarly, Gobakis *et al*.^16^ compared the performance of three types of ANN architectures (Cascade, Elman, and Feedforward) for UHI prediction using ambient air temperature and global solar radiation data collected from 14 meteorological stations in Athens. The study results indicated Elman ANN as the optimum neural-network architecture for predicting UHI.

While ANN algorithms generally provide better fitted models than conventional statistical techniques, mainly owing to their ability to learn and mimic highly complex non-linear data, existing studies have mostly employed shallow ANNs (i.e., neural networks with a single hidden layer) to study UHI. However, ANN are prone to overfitting and often face problems related to generalizability^17^. To address this issue, we developed two types of deep neural network (DNN) models that predict and quantify the UHI effect in Seoul; a temporal and a spatial model. Spatial-pattern modeling attempts to explain the UHI phenomenon using geographic indicators (e.g., land-cover types), whereas temporal modeling uses meteorological indicators (e.g., air temperature) to explain UHI distribution. Spatial patterns have been used to explain several issues in urban environments^12,13,18,19^. The main difference between the temporal UHI-model and the spatial UHI- model developed in the current study is that the spatial UHI-model explains how urban factors affect the manifestation of UHI in urban areas whereas the temporal UHI-model explains how meteorological elements affect the manifestation of UHI in urban areas.

There are also several studies that report on the UHI phenomenon in Seoul specifically^20–23^. Although the analysis of UHI intensity in Seoul has been reported previously, the existing studies used either single-year meteorological data or data collected from only a few AWSs. As such, analyses from previously developed UHI-predictive models for Seoul, such as those reported by Lee *et al*.^20^ and Kim and Baik^21^, are likely to be limited, given that they did not use multiyear meteorological data, which are essential in developing accurate UHI-predictive models. The studies that did use multiyear meteorological data in model development, such as Kim and Baik^19^, did so with data collected from relatively few AWSs, which also limited the breadth of their analysis. Consequently, to overcome the shortcomings of previous studies concerning UHI in Seoul, we used multi-year meteorological data collected over a period of 9 years (2009–2017) from 54 AWSs located across Seoul. The use of multi-year datasets in the analysis of UHI is likely to provide better results than using single-year datasets because data from a single particular year is unlikely to be a proper representative of typical weather conditions within a region. There are certain climatic events (i.e. increased heat waves) that occur in a particular year but which are not common occurrences in the region of interest. Moreover, data from 26 of the 54 AWSs is new data being used to study UHI in Seoul for the first time. It is also important to realize that UHI behavior in a given area is likely to change over time due to factors such as development and changes in land use. Therefore, the analysis of UHI in a given area should be based on recent meteorological and land-use data. Thus, unlike previous studies concerning UHI in Seoul, particularly those involving the development of predictive models, our study uses the most recent meteorological and land-use data, and therefore, provides insights into the current status of the UHI phenomenon in Seoul. Furthermore, UHI effects are commonly assessed using UHI intensity as an indicator^24^. Although UHI intensity is a useful and accurate indicator of UHI variations within a time frame, it has been reported to underestimate UHI levels at peak solar time or during the daytime^25^. Moreover, UHI intensity is a measure of UHI at a specific time and therefore inadequate to assess the cumulative effects of UHI. Consequently, we develop and propose the use of a new metric, UHI-hours, that computes the total number of hours that UHI exists in a given area. Such a measure is useful in quantifying the cumulative effects of UHI over a prolonged period of time. For instance, UHI-hours can be used to show the potential correlations between UHI and reduced productivity of outdoor workers, mortality rates in summertime, annual building energy consumption etc.

Conclusively, the aims of the current study are twofold; The first aim was to develop and propose a new metric to assist in quantifying the cumulative effects of UHI. The second aim was to develop data-driven models that can be used to predict UHI levels using either temporal data (e.g. meteorological factors) or data related to the spatial characteristics of a given area (e.g. land use data).

## Results

### Relations among UHI intensity, UHI-hours, and air temperature

**Figure 1:**
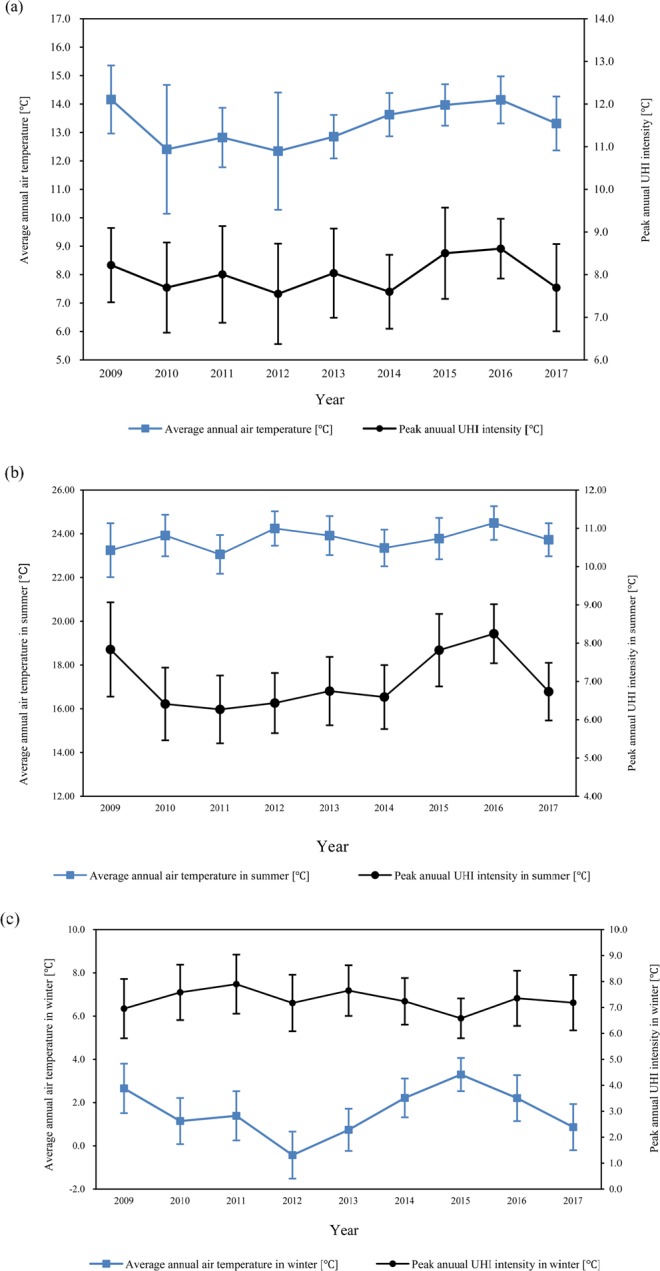
Changes in average ambient temperature and seasonal peak UHI intensity: (**a**) Average annual air temperature and peak annual UHI intensity. (**b**) Average ambient temperature and seasonal peak UHI intensity in summer (**c**) Average ambient temperature and seasonal peak UHI intensity in winter; (the error bars indicate standard deviations).

The trends in our data can be described in two distinct phases; changes in UHI intensity and air temperature over the years and correlations between UHI intensity and air temperature over the years. As shown in Fig. 1(a), our results show no clear trends in air temperature over the years. The air temperature tended to increase and decrease over the years in no particular order. For example, while the average air temperature was 14.2 °C in 2009, it was 12.3 °C in 2012 and 14.1 °C in 2016. This same trend is observed for UHI intensity. However, air temperature itself was correlated with UHI intensity. The years with high average air temperatures are the same years which experienced high average UHI intensity and vice-versa. For a detailed analysis, the relation between the average ambient measured temperature and peak measured UHI intensity was determined for two seasons: summer and winter. Based on existing empirical studies^26^, the summer season in Seoul is considered to be from April 30 to September 7, and the winter is considered to be from November 1 to February 28.

Furthermore, as presented in Fig. 1(b,c), the average seasonal ambient temperature and peak seasonal UHI intensity were not, in general, proportional to each other, except during certain periods. From 2013 to 2017, the average ambient temperature during the summer season decreased from 23.9 °C in 2013 to 23.3 °C in 2014; then, it steadily increased to 24.5 °C in 2016 and decreased again to 23.8 °C in 2017. During the same period, the summer peak UHI intensity decreased from 6.8 °C in 2013 to 6.6 °C in 2014; then, it increased to 8.3 °C in 2016 and decreased again to 6.7 °C in 2017. Thus, for the period 2013–2017, the summer peak measured UHI intensity was proportional to the average ambient temperature in the summer season. However, during 2009–2010 and 2012–2013, while the average summer ambient temperatures varied within 23.2–23.9 °C and 24.2–23.9 °C, respectively, the summer peak UHI intensity changed from 7.8 to 6.4 °C and from 6.4 to 6.8 °C, showing no clear relation between temperature and UHI for the periods 2009–2010 and 2012–2013.

In the winter season during 2010–2013, the average ambient temperature increased from 1.1 °C in 2010 to 1.4 °C in 2011, and then decreased to −0.4 °C in 2012 and increased again to 0.7 °C in 2013. The same trend of a repeated increase and decrease was observed in the peak UHI intensity, which changed from 7.6 °C to 7.9 °C, to 7.2 °C, and to 7.7 °C, thereby showing a correlated relation between air temperature and UHI intensity during winters. During 2014–2017, the average winter ambient temperature increased from 2.2 °C to 3.3 °C, and then decreased to 1.1 °C. During the same period, the peak UHI intensity repeatedly increased and decreased, from 7.2 °C to 6.6 °C, to 7.4 °C, and to 7.2 °C. Because we found no clear relation between air temperature and UHI intensity, and since UHI-hours is a better representation of cumulated heat islands, we conducted further analyses to see if there was any relationship between seasonal air temperature and UHI-hours.

**Figure 2:**
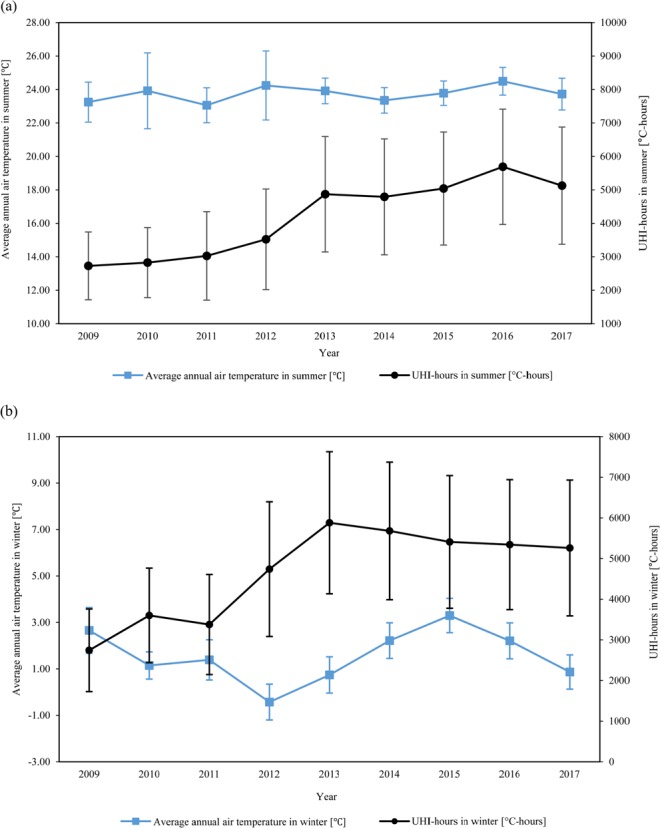
Changes in seasonal average ambient temperature and seasonal peak UHI-hours: (**a**) summer, (**b**) winter; (the error bars indicate standard deviations).

Earlier, we explained that UHI-hours is the sum of hours when the UHI intensity is above 0 deg C for a certain period of time. Comparing UHI-hours and UHI-intensity therefore, our results showed that the average seasonal temperature is more correlated with UHI-hours than UHI intensity. For instance, when the summer UHI-hours increased from 4972 °C-h to 5888 °C-h between 2014 and 2016, the average ambient measured temperature also increased as indicated in Fig. 2(a). However, when UHI-hours decreased to 5289 °C-h in 2017, the average summertime measured ambient temperature also tended to decrease. In winter, except for the two years from 2014 to 2015, the average wintertime ambient temperature showed a correlated relation with wintertime UHI-hours as shown in Fig. 2(b).

### Relations among spectral radiance, surface albedo, and UHI-hours

**Figure 3:**
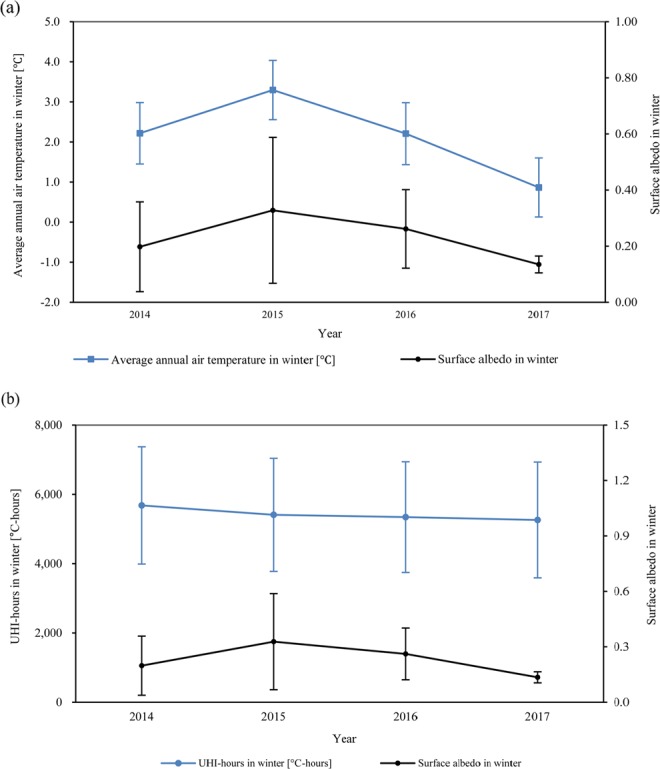
Wintertime changes in surface albedo with (**a**) average ambient temperature and (**b**) UHI-hours; (the error bars indicate standard deviations).

We analyzed the relations among spectral radiance, surface albedo, and seasonal air temperature as well as those among spectral radiance, surface albedo, and seasonal UHI-hours for the period between 2014 and 2017 (Fig. 3). During 2014–17, the results show that there was a potential correlation between the average winter surface albedo both the average ambient temperature as shown in Fig. 3(a) and UHI-hours as shown in Fig. 3(b). The winter surface albedo increased from 0.20 to 0.33 and then decreased to 0.13. This trend was similar to that of the average winter ambient temperature in the same period. In addition, the winter UHI-hours during the same period steadily decreased from 5680 °C-h in 2014 to 5260 °C-h in 2017. During 2015–2017, the changes in UHI-hours in the winter season were correlated to those in winter surface albedo. Besides conveying simple weather information over time, surface albedo is also a surrogate for land-surface changes over time. As such, our results suggest that both the seasonal average ambient temperature and UHI-hours are influenced by the changes in urban morphology (e.g., changes in land-surface type). However, we found no substantial relation between spectral radiance and average ambient temperature or peak UHI-hours.

### Temporal UHI model

**Figure 4:**
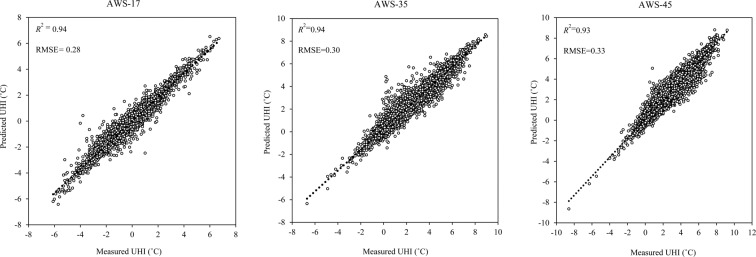
Performance of DNN temporal UHI model. [AWS, automatic weather station; *R*^2^, coefficient of determination; RMSE, root-mean-square error; UHI, urban heat intensity].

We developed a temporal UHI model to use with Seoul AWS data, showing maximum, minimum, and median temperatures (AWS-17, AWS-35, and AWS-45, respectively), using a deep-learning method. Here, model performance results are presented for both the training and the validation datasets. The prediction models for UHI variations for the three AWSs showed that, for the training dataset, the model for AWS-35 had the highest *R*^2^ (0.98) and lowest RMSE (0.26), the model for AWS-45 had the next highest *R*^2^ (0.97) and next lowest RMSE (0.27), and that for AWS-17 had the lowest *R*^2^ (0.96) and the highest RMSE (0.29). In case of the validation dataset, the models for AWS-17 and AWS-35 had the same *R*^2^ (0.94) and that for AWS-45 had *R*^2^ = 0.93. The model developed using data from AWS-17 had the lowest RMSE (0.29), followed by the model for AWS-35 (RMSE = 0.41), and that for AWS-45 (RMSE = 0.44). Also, we used the combined training and validation datasets to predict the UHI distribution for each location selected based on the actual UHI intensity obtained through the inverse-distance-weighted interpolation method. Generally, the prediction model developed with data from AWS-17, which also showed the maximum annual UHI-hours, produced the highest *R*^2^ and lowest RMSE values, whereas that developed with data from AWS-45, which was associated with median annual UHI-hours, produced the lowest *R*^2^ and highest RMSE (Fig. 4).

### Spatial UHI model

We also developed a deep-learning model based on urban spatial information to predict UHI distribution during the winter and summer seasons. This model was developed to predict UHI intensity and UHI-hours for the entire area of Seoul and to quantitatively analyze the effect of urban spatial elements on UHI and UHI-hours distribution. Our results showed that prediction models for UHI-hours generally had a higher *R*^2^ value than those for UHI intensity. Both UHI intensity and UHI-hours models showed a higher *R*^2^ value and a lower RMSE value in summer than in winter. In summer, the models showed *R*^2^ values of 0.98 and 0.99 for UHI intensity and UHI-hours prediction, respectively. In winter, the model had the same *R*^2^ value of 0.99 for both UHI intensity and UHI-hours prediction. Furthermore, the model for predicting UHI intensity had an RMSE value of 0.08 in summer and 0.09 in winter, while that for UHI-hours had an RMSE value 145.22 in summer and 176.68 in winter. Table 1 shows the performance of the developed models for the prediction of UHI intensity and UHI-hours according to each season.

**Table 1:** Performance of deep-learning prediction models based on spatial data.

Spatial model	Season	*R*^2^	RMSE
UHI intensity UHI intensity	Summer	0.98	0.08
	Winter	0.99	0.09
UHI-hours UHI-hours	Summer	0.99	145.52
	Winter	0.99	170.68

*R*^2^, coefficient of determination; RMSE, root-mean-square error; UHI-hours, urban heat island hours; UHI intensity, urban heat island intensity.

#### Relative importance of predictive factors for UHI

We analyzed the relative importance of physical and environmental factors affecting UHI using a deep-learning approach and UHI-hours as an indicator. We applied the Gedeon method^26^, which uses functional analysis of the weighted matrix of a trained neural-network model to determine the behavioral significance of the model features in the hidden neurons. We found that in summer, the green-area, road-area, cropland-area, and bare-area ratios were the most important factors influencing UHI-hours, whereas spectral radiance and surface albedo had less impact. In winter, the green-area and road-area ratios were the most important factors affecting UHI-hours.

We also found that in both summer and winter, the green-area, road-area, and bare-area ratios were the most important factors affecting UHI-hours, whereas the sky-view factor (SVF) and the footprint were the least important factors affecting UHI-hours. However, the total floor-area ratio had a higher impact on UHI-hours than the footprint ratio.

Factors such as water-area ratio, spectral radiance, and surface albedo showed different degrees of influence on UHI-hours according to season. In summer, the water-area ratio was an important factor affecting UHI-hours. However, its influence was less significant during winter. In addition, the impact of spectral radiance and surface albedo on UHI-hours was more pronounced in winter than summer.

## Discussion

The present study provides a comprehensive analysis of the UHI phenomenon in Seoul city. We use a large dataset of meteorological elements (i.e. 9-year climatic data) and land use data (i.e. 3-year data) collected at short time intervals. Thus, because of the large amount of data collected and the intervals at which they were collected, including the data used for the first time in the analysis of UHI in Seoul, our study provides a deeper understanding of which factors are more likely to influence UHI behavior in an urban environment. For example, the data used in previous studies for UHI prediction were mostly collected at relatively long time intervals (3 or 6 h) compared to the data used in the present study. For instance, Kim and Baik^23^ used data from a single AWS collected at 6-h intervals to predict UHI variations in Seoul. Similarly, Pakarnseree *et al*.^27^ used climate data collected over a 5-year period at 3-h intervals to analyze UHI patterns in Bangkok. In our study, however, we used climate data collected over a 9-year period at 1-h intervals, thus allowing for a more-resolved analysis of UHI distribution in Seoul. This is because using data recorded in short time intervals (e.g. 1 hour) over that recorded in long time intervals (e.g. 3 hours), especially when dealing with dynamic physical elements such as air temperature, enables us to capture the sudden changes that occur in very short time spans which might be quite influential when predicting UHI.

Similarly, compared to previous studies, the data used in the present study was collected from an extensive network of stations. For example, Tan *et al*.^28^ used data from 11 AWSs to examine the UHI effect on human health in Shanghai. Similarly, Li *et al*.^29^ used data from four AWSs to analyze the relation between UHI intensity and summertime air pollution in Berlin. However, in the present study, we used data from 54 AWSs spread across Seoul city. The use of data collected from an extensive network of stations (i.e. spread over Seoul city) is important because it ensures that the models are trained on a heterogeneous dataset of the factors affecting the manifestation of UHI and consequently resulting in a better understanding of how different factors influence UHI behavior. For example, the 54 AWSs are spread across spots/areas with different characteristics. These different characteristics impact on the manifestation of UHI differently, especially those related to land use. By using data from an extensive network of stations therefore we ensured that the values of model variables are represented in diverse ranges and thus making the developed models more generalizable compared to the models developed in previous studies. To compare the coverage of the AWS used in our study and those from previous studies, we calculated and compared the area represented by each AWS between the abovementioned studies and our study. We found that the areas of the previously studied cities of Shanghai^28^ and Berlin^29^ and of Seoul (i.e. our study) are approximately 6340 km^2^, 900 km^2^, and 605 km^2^, respectively. As such, while the surface area represented by each AWS in these two previous studies was 576 km^2^ (Shanghai) and 225 km^2^ (Berlin), the surface area represented by each AWS in our study was 11 km^2^. This implies that the models developed in the present study are more representative of our subject area (i.e. Seoul) than those developed by other studies.

Another contribution of our study deals with quantifying and measuring UHI variation. To quantify UHI effects, many researchers have used UHI intensity as a metric. Although UHI intensity is accurate in representing urban heating during the nighttime and morning hours, it underrepresents urban heating at peak solar hours or in periods dominated by solar heating^25^. Consequently, we developed and proposed the use of a new metric, UHI-hours, to quantify UHI. UHI-hours allows for the cumulative quantification of UHI patterns in a small local area or UHI patterns for a specific season. Our results indicated that UHI-hours is a more accurate metric than UHI intensity for assessing seasonal UHI behavior. Consequently, UHI-hours is more likely to provide a better measure of the cumulative effects of UHI within an urban environment. For example, UHI-hours is more likely to be correlated with the overall reduced productivity of outdoor workers than UHI-intensity. Similarly, UHI-hours is likely to be better correlated with mortality rates due to heat stress in the summertime than UHI-intensity. This is primarily because the effect of heat exposure on the body builds up over time which in turn gradually starts affecting different physiological processes of the body. As such, UHI should be quantified as a cumulative element rather than a distinct value such as in the case of UHI-intensity.

Our research also improves on the predictive models previously developed to study and predict UHI behavioral patterns. The earlier studies mostly applied ANN and time-series modeling techniques to predict UHI intensity distribution. For example, Lee *et al*.^20^ developed an ANN model to predict UHI intensity in Seoul using climate data from 28 AWSs and urban spatial characteristics. Kim and Baik^21^ also developed an ANN model to predict UHI intensity in Seoul, which they based on the four factors of wind speed, relative humidity, cloudiness, and UHI intensity from the previous day. However, these two studies developed shallow ANN models, which are prone to overfitting and are not generally conducive to generalization^17^. To address this issue, we developed DNN models, which are less prone to overfitting and generally perform better on new data as compared to shallow ANN models. This implies that the models developed in the present study can be successfully used to predict UHI in other parts of Korea.

**Figure 5:**
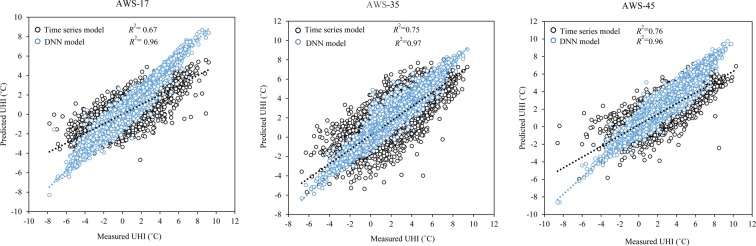
Comparative performance between the UHI-predictive models developed using the DNN algorithms and performance of UHI-predictive models developed using time-series algorithm models.

Furthermore, many studies have often used time-series models to study UHI^30–32^. Consequently, we developed a time-series model, based on ARIMA, for use as a benchmark model to assess the performance of the developed temporal UHI model relative to previously developed models. The algorithm used to develop the time-series models is based on autoregressive integrated moving average (ARIMA) modeling^30,31^, which allows for the prediction of future data based on past data and the relations among the error factors. Our results showed that DNN models had a better predictive ability than the time-series models. The overall *R*² value was significantly higher for the DNN model than for the time-series model (Fig. 5); the difference in the *R*² value ranged from 0.19 and 0.29. As such, our results suggested that DNN-based models are likely to be more beneficial (i.e. in terms of predictive accuracy) in studies concerning UHI than models based on time series modelling techniques.

We also used the developed spatial model to determine the relative importance of the physical and environmental factors influencing UHI formation. The results of its application indicated that green- and road-area proportions are the main factors that influence UHI formation in Seoul in both the summer and winter seasons. This finding is consistent with those of previous studies, such as Li *et al*.^33^, who reported that UHI was highly correlated with the type of land surfaces present in an area, and Weng *et al*.^34^, who revealed the effect of green areas on the control of surface temperatures. In addition, our results indicate that the total-floor-area ratio is an important factor influencing UHI distribution. This finding is also consistent with reports from previous studies, such as that by Chun and Guldmann^12^, who reported the influence of building and road density on UHI in central urban areas. Consequently, the results obtained in the present study suggest that increasing the green areas and reducing impervious surfaces in urban areas are essential in combating adverse UHI effects. Also, the proposed method allows for the understanding of the impact of greenery under variable boundary conditions and for different synoptic systems. Additionally, the information provided here regarding the hierarchical ranking of the land use factors that are most likely to affect UHI can be leveraged by policy makers when drafting policies to mitigate the effects of UHI in urban climates.

While the present study presents useful information regarding the current status of the UHI phenomenon in Seoul city and subsequently develops accurate UHI-predictive models, it still faced certain limitations. One of the limitations deals with the variety of variables used to analyze UHI. Previous studies have shown that UHI can be influenced by a range of variables such as solar radiation^35^, cloud cover^36^, rainfall^37^ etc. However, data related to such variables for Seoul city was not available from the AWS at the time of this study. Also, data related to the spatial characteristics of Seoul city was obtained from publicly available government databases which were also limited in variety. As such, we were unable to consider the contribution of many variables in our analysis. Future studies therefore should assess the contribution of several other variables on UHI.

Another limitation of our study deals with the radius at which the land use variations were assessed. The variations in land use were assessed in a 1-km radius within each AWS. However, using shorter distances (100 m–500 m) is likely to provide better results than those presented in the current study. Future studies therefore should consider using shorter distances when analyzing variations in land use.

Also, the data used to assess the influence of surface albedo and spectral radiance on UHI was based on data collected over a 4-year period (2013–2014). This is because the satellite used to collect “surface albedo” and “spectral radiance” data only started operating in 2013. To reveal further correlations among the said variables therefore, we suggest that future research studies use a larger dataset containing spectral radiance and surface albedo data collected over a longer period of time.

Furthermore, as we explained earlier, we develop two separate models for predicting UHI; a temporal UHI-predictive model and a spatial UHI-predictive model. The purpose for developing two separate models was so that we provide a model (i.e. spatial model) that explains how urban features affect the manifestation of UHI in urban areas and a separate model (i.e. temporal model) that explains how meteorological elements affect the manifestation of UHI. However, a model that explains the behavior of UHI using a combination of urban factors and meteorological elements is still necessary. Such a model would require that all the variables used in the training process be collected over the same time period, especially if supervised learning methods are to be employed in model development. This is a major reason that prevented the current study from developing a model that considered both the temporal and spatial factors influencing UHI; the available spatial and temporal data were recorded over different periods. Future research studies should endeavor to obtain spatial and temporal data collected over the same period of time and subsequently develop models able to explain the behavior of UHI based on a combination of both the meteorological and urban texture elements.

## Conclusion

UHI causes several issues related to health, energy consumption, comfort, etc., in urban areas. Also, the increase in urban development projects as well as the effects of climate change are likely to result in increased UHI in the future. Consequently, to mitigate the effects of UHI, proper and accurate tools to analyze and predict UHI behavior are of paramount importance. In the current study, we show that deep learning methods are useful tools that can be used to accurately analyze and predict UHI behavior in urban areas. We also show to what extent different urban texture elements are correlated with UHI. Such information can be leveraged by policymakers when drafting mitigation plans for UHI. Furthermore, the new index, UHI-hours, developed in the present paper provides a more accurate measure of the cumulative effects of UHI than other commonly used indices such as UHI intensity.

## Methodology

### Meteorological data

**Figure 6:**
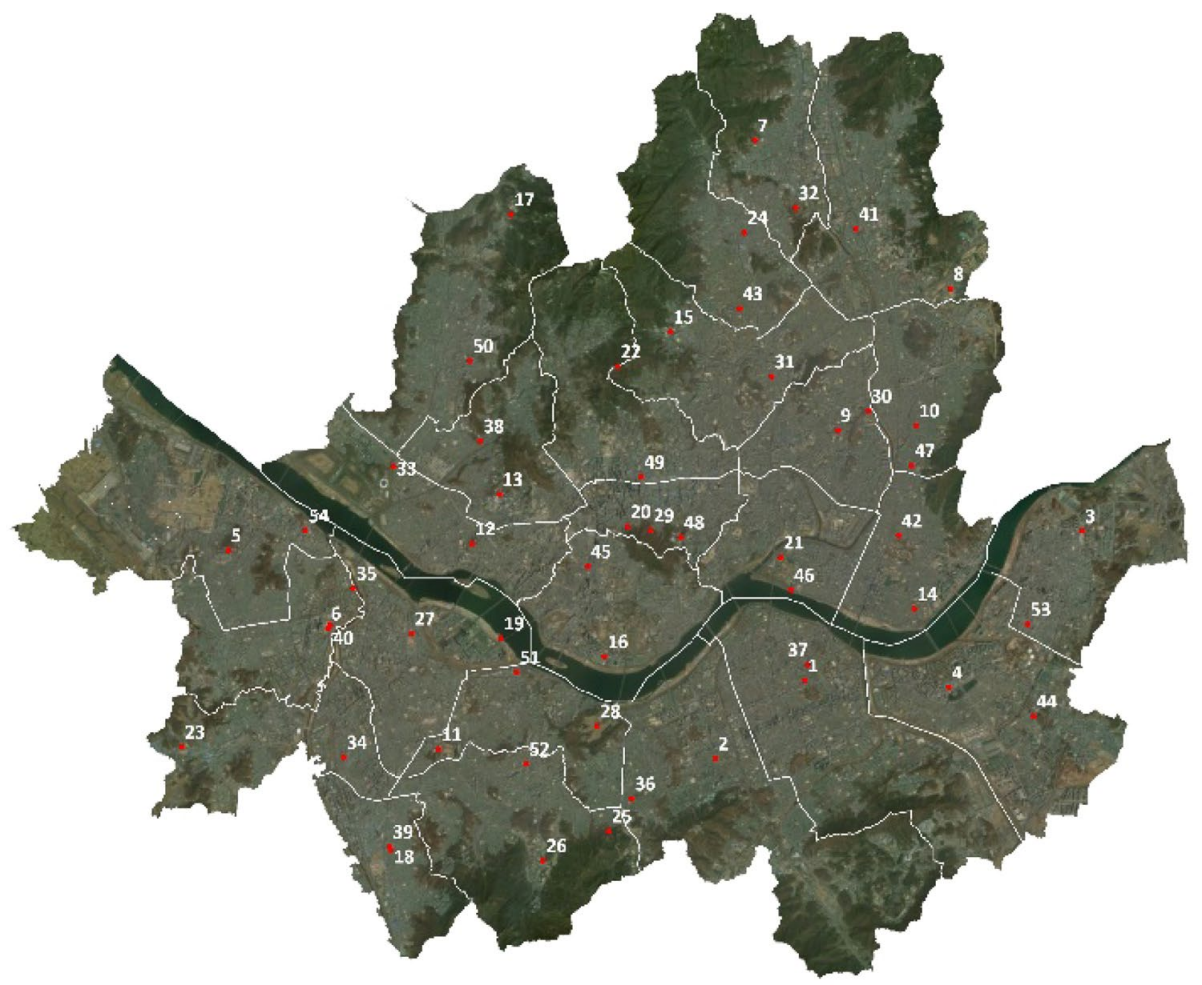
Locations of the 54 Automatic Weather Stations (AWS) in Seoul city. (Figure generated using ArcMap, version 10.6.1, https://desktop.arcgis.com/en/arcmap/).

The meteorological data used in this study were obtained from 54 AWSs in Seoul, of which 28 are operated by the KMA and 26 are operated by the Seoul city office as presented Fig. 6. The AWSs collect meteorological data in 1-min intervals. For the present study, we gathered air-temperature, wind-direction, and wind-velocity data measured at the 54 AWSs for a period of 9 years.

### Physical and environmental factors

The factors that contribute to UHI effects have been reported in several previous studies^19,38–49^. These factors can be grouped into two categories, physical and environmental. In our study, data for both environmental and physical factors were gathered within a 1-km radius of each AWS.

Physical factors include sky view factor^38^, total floor areas of entire buildings^39^, area covered with green vegetation^40^, building footprints, area covered with water and crops, and bare land area^41–43^. In our study, we obtained information related to physical factors from publicly available data on land-use patterns and building-management systems from a Geographic Information System (GIS) data from ministry of Land, Infrastructure and Transport Korea^38,39^. Environmental factors include spectral radiance^44,45^ and surface albedo^45–47^. We collected data related to environmental factors from the metadata of the Landsat 8 remote-sensing satellite^46,50^.

Landsat 8 (Operational Land Imager and Thermal Infrared Sensor) captures images with an approximate extent of 170 km north–south by 185 km east–west^50^. It takes about 15 days to circumnavigate the entire globe, and thus, returns to the same area about every 16 days^51^. Data from the Landsat satellite are easy to obtain and have been reported to be accurate^52^. Therefore, we used data collected by the Landsat 8 satellite for the period between 2014 and 2017 in 32-day intervals. However, the Landsat 8 satellite metadata for Seoul contain data for the entire Seoul area, whereas we needed spectral-radiance which is the amount of radiant flux from the sun per unit surface on land considering the spectrum of light from the sun^53^ and surface-albedo data for a 1-km radius from each AWS. To obtain such urban radiance data corresponding to each AWS, we used a formula provided by the USGS^52^:

1 $${L}_{\lambda }={L}_{{\rm{\min }}(\lambda )}+({L}_{{\rm{\max }}(\lambda )}-{L}_{{\rm{\min }}(\lambda )})\ast {Q}_{DN}/{Q}_{max}$$

where L_λ_ is the spectral radiance received by the sensor, Q_max_ is the maximum DN of the IR bnad and L_max(λ)_ and L_min(λ)_ are the maximum and minimum detected spectral radiance^53^.

Surface albedo was computed from Landsat metadata, after converting DN value to the Top of atmosphere(TOA) reflectance values, the values of surface albedo ranging from 0 to 1

2 $${\rm{\rho }}\,\lambda {\prime} ={M}_{\rho }{Q}_{cal}+{A}_{\rho }$$

TOA reflectance was computed from Landsat digital number(DN) data. ρ*λ*′ is TOA planetary reflectance, *M*_*ρ*_ is band-specific multiplicative rescaling factor, *Q*_*cal*_ is quantized and calibrated standard product pixel values, *A*_*ρ*_ is band-specific additive rescaling factor^54^.

3 $${\rm{\alpha }}=\frac{((0.356\ast \rho 2)+(0.130\ast \rho 4)+(0.373\ast \rho 5)+(0.085\ast \rho 6)+(0.072\ast \rho 7)-0.018)}{1.016}$$

### Concept of UHI-hours

We developed a new assessment metric to quantify the UHI effect. The proposed metric, UHI-hours, follows the same concept as the “degree days” coefficient^55^ used to quantify the energy demand in buildings. Degree days are calculated as the absolute difference between a reference temperature and the daily average temperature and represent a versatile climatic indicator commonly used in building energy performance analysis^56^. UHI-hours indicates to what degree the average temperature differs from the expected temperature (i.e., design temperature), and consequently, how much energy is demanded. Degree days can also be used to measure differences in regional temperature profiles^57^ or to UHI-hours estimate to what degree the ambient-temperature difference between a given urban area and a rural area differs from 0. In our study, we selected the reference rural area based on a previous study on UHI in Seoul (i.e. Neunggok station). Neunggok is at the same latitude with Seoul and meets the criteria dictated by the World Meteorological Organization (WMO)^58^ for choosing reference rural environments in UHI studies. Ultimately, the UHI-hours concept can also be used to gauge cumulative UHI intensity from season to season UHI-hours, thus allowing for a more efficient quantification of UHI for a small area or for a given season. UHI-hours is calculated by

4 $$UHI-hours={\sum }_{i=1}^{n}{t}_{k}({T}_{urban}-{T}_{rural})hour$$

where *T*_*urban*_ and *T*_*rural*_ are the average air temperatures of urban and rural environments, respectively, and constant term *t*_*k*_ = 1 if (*T*_*urban*_ − *T*_*rural*_) > 0 and *t*_*k*_ = 0 if (*T*_*urban*_ − *T*_*rural*_) ≤ 0.

### Deep-learning methods

**Figure 7:**
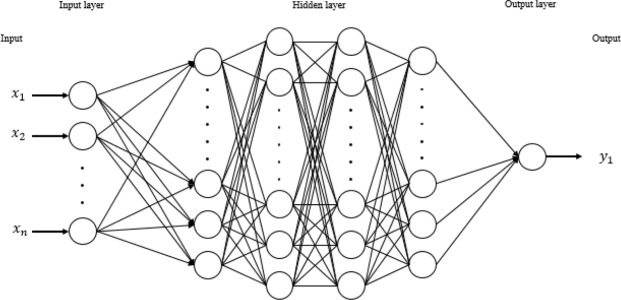
Schematic diagram of an Artificial Neural Network (ANN) architecture.

We developed two deep-learning models that predict UHI distribution—a temporal and a spatial UHI model. The difference between the two lies in the input variables used to explain UHI in each model. The input variables used to develop the temporal UHI model are mainly time-dependent meteorological factors (i.e. date, time, air temperature, wind speed, and wind direction) whereas the spatial UHI model is based on the physical and environmental factors (i.e. sky view factor, total floor areas of entire buildings, area covered with green vegetation, building footprints, area covered with water and crops, bare land area, spectral radiance and surface albedo). The deep-learning algorithm used to develop the models is based on a multilayer feedforward ANN concept^59^. A typical multilayer feedforward ANN model is composed of several layers of interconnected units, called neurons (Fig. 7). In the prediction model, the weighted combination of input signals (represented by *ω*_*i*_ and *x*_*i*_) is aggregated, and then, the output signal f(α) is transmitted by the connected basic neuron, as

5 $$\alpha ={\sum }_{i=1}^{n}{\omega }_{i}{x}_{i}+b$$

where *ω*_*i*_ is the weight, *x*_*i*_ is the dependent variable, *b* is a constant, and *α* is the output variable.

In the feedforward system, the bias units are included in each of the non-output layers of the network. The weights that connect neurons and bias with other neurons affect the output of the whole network. Learning occurs when these weights are adjusted to minimize errors in the results. In our models, the loss function is based on the mean square error value (Eq. (5)), and the objective in each training example (*j* in Eq. (5)) is to minimize the loss function. Basically, *W*_*i*_ denotes the weight matrix connecting layers *i* and *i* + 1 for a network of *N* layers. *B*_*i*_ also denotes the column vector of biases for layer *i* + 1. *t*^(*i*)^ and *o*^(*j*)^ are the predicted and actual values, respectively, for training example *j*. The activation function used is the rectifier function with dropout; its role is to ensure that each neuron chooses the largest output from separate channels with its own weights and bias values. During the process of model training, we used several activation functions to see which one provided optimum model performance. Our results showed the rectifier function to provide the best model performance. This outcome is also in line with previous studies^60^ which report the rectifier function as being superior to other activation functions.

For the temporal model, hourly data of ambient temperature, wind direction, and wind speed measured during 2009–2017 were used as features in the development of the prediction model. The data from 2014 to 2015, when the land-cover data were also determined, were used as the validation dataset, and the remaining data (2009–2013 and 2016–2017) were used as the training dataset. While developing the model, the number of layers and neurons were consistently increased until there was no further significant change in *R*^2^ and root-mean-square error (RMSE) values. Thus, a total of five hidden layers were used, and the number of neurons was between 200 and 400 per layer. For the spatial model, the urban spatial data used to train the model (i.e., physical and environmental factors) was collected in 2014, and the environmental data (i.e., surface albedo and spectral radiance) derived from the same dataset. This prediction model was constructed by identifying the numbers of layers and neurons that produced the maximum possible *R*^2^ and the lowest possible RMSE value, as was also done for the temporal UHI model discussed above. Accordingly, a total of three layers were used and the number of neurons was between 200 and 400 per layer.

6 $${\rm{L}}(W,B|j)=\frac{1}{2}{\Vert {t}^{(i)}-{o}^{(j)}\Vert }_{2}^{2}$$

7 $${\rm{L}}{\prime} (W,B|j)=L(W,B|j)+{\lambda }_{1}{R}_{1}(W,B|j)+{\lambda }_{2}{R}_{2}(W,B|j)$$

To avoid overfitting, we used regularization variables, ${\ell }_{1}$ (L1: Lasso) and ${\ell }_{2}$ (L2: Ridge), to modify the loss function, and thus, minimize loss. For ${\ell }_{1}$ regularization (Eq. (6)), *R*_1_(*W*, *B*|*j*) is the sum of all ${\ell }_{1}$ norms for the weights and biases in the network, and ${\ell }_{2}$ regularization *R*_2_(*W*, *B*|*j*) is the sum of squares of all weights and biases in the network. The network weights for ${\ell }_{2}$ regularization are scaled toward 0. In addition, the algorithms share the same global parameters but learn different models through each training session. Therefore, we used the dropout function to allow an exponentially large number of models to be averaged as an ensemble. This reduces the likelihood of overfitting and improves model generalization.

### Time-series modeling

To compare the previous studies^61,62^, which used time-series modeling to predict UHI, with our study, we used the same data to develop a time-series model. We applied an autoregressive integrated moving average (ARIMA), a class of statistical models used for forecasting time-series data. In ARIMA, the variable of interest is assumed to be a function of its past observations and random errors. The model is categorized as ARIMA (*p*, *d*, *q*), where *p* denotes the autoregressive parts of the dataset, *d* refers to integrated parts of the dataset, and *q* represents moving-average parts of the dataset. A typical ARIMA model^32^ can be represented as

8 $$\begin{array}{ccc}{y}_{t} & = & {\theta }_{0}+{\phi }_{1}{y}_{t-1}+{\phi }_{2}{y}_{t-2}+\cdot \cdot \cdot +{\phi }_{p}{y}_{t-p}\\  &  & +\,{{\rm{\varepsilon }}}_{t}-{\theta }_{1}{{\rm{\varepsilon }}}_{t-1}-{\theta }_{2}{{\rm{\varepsilon }}}_{t-2}-\cdot \cdot \cdot -\,{\theta }_{q}{{\rm{\varepsilon }}}_{t-q,}\end{array}$$

where *y*_*t*_ is our variable of interest; *ε*_*t*_ is a random error at specific time *t*; *ϕ*_1_, *ϕ*_2_, ….*ϕ*_*p*_ and *θ*_0_, *θ*_1_, *θ*_2,_ ….*θ*_*q*_ are model parameters; *p* and *q* are all non-negative integers; and errors *ε*_*t*_ are assumed to have a mean of zero and constant variance.

## Data Availability

The datasets generated and analysed during the current study are available from the corresponding author on reasonable request and with permission of Korea Meteorological Administration and Seoul Metropolitan Government.
